# The Peripheral Defocus Designed Spectacle Lenses Might Increase Astigmatism in Myopic Children

**DOI:** 10.1167/tvst.14.3.8

**Published:** 2025-03-11

**Authors:** Wenyan Xu, Xiaoman Li, Jianing Zhang, Hongyi Li, Xuewen Ding, Xiaoyue Hu, Xinyue Quan, Yue Su, Fan Lu, Jie Chen

**Affiliations:** 1 National Clinical Research Center for Ocular Diseases, Eye Hospital, Wenzhou Medical University, Wenzhou, Zhejiang, China

**Keywords:** peripheral defocus spectacle lenses, astigmatism, myopia

## Abstract

**Purpose:**

This study aims to explore the impact of wearing peripheral defocus spectacle lenses (PDSL) on cylindrical refractive error (CYL) in myopic children.

**Methods:**

This study included 1057 myopic children and divided the participants into three groups: the HAL group (spectacle lens with highly aspherical lenslets), the MPV group (spectacle lens based on manipulating peripheral vision), and a control group (without myopia control interventions). The study analyzed the effect of wearing PDSL on changes in spherical equivalent refraction, CYL, and corneal astigmatism (CA). The mediating effect between changes in spherical refractive errors (SPH) and CYL was also investigated.

**Results:**

Compared to the control group (0.05 ± 0.33 D), the annual CYL progression was faster in the HAL group (−0.15 ± 0.33 D, *P* < 0.001) and the MPV group (−0.09 ± 0.27 D, *P* = 0.019). More children in the HAL group had an annual CYL progression ≥0.50 D (HAL: 23.6%, Control: 16.2%, *P* = 0.012). The annual CYL and CA progression were consistent within the PDSL groups (HAL: *P* = 0.677, MPV: *P* = 0.683). The total effect of CYL progression in the HAL group was primarily due to direct induction from wearing HAL and indirect induction through the SPH control effect.

**Conclusions:**

The application of PDSL could cause increase in astigmatism in myopic children, which could mainly be contributed to cornea astigmatism change.

**Translational Relevance:**

PDSL may passively affect the anterior ocular biomechanics during myopia control, leading to an increase in astigmatism.

## Introduction

Myopia prevalence is increasing worldwide. It is estimated that half of the world's population (4758 million) would have myopia by 2050, of which 938 million are high myopia.^1^ Myopia prevalence in school-aged children in Eastern Asia is significantly higher than other areas.^2^^–^^4^

Research in animal models have shown peripheral visual signals could dominate central refractive development.^5^ By altering the defocus state of the peripheral retina, significant impacts on changes in refractive error and axial length can be achieved.^6^ Specifically, myopic defocus can slow eye growth, whereas hyperopic defocus accelerates it.^7^ Based on this principle, peripheral defocus spectacle lenses (PDSL) have been developed and have been demonstrated to be effective in slowing myopia progression.^8^^–^^13^ The principle function of defocus spectacles is to create myopic defocus on the peripheral retina while concurrently providing distance vision correction in the central zone. There are various designs of PDSL, including spectacle lenses based on manipulating peripheral vision (Myovision; Zeiss, Oberkochen, Germany)^14^^,^^15^ and spectacle lenses with highly aspherical lenslets (Stellest; Essilor, Charenton-le-Pont, France).^16^^–^^18^

Myovision lenses (MPV) are very early myopia control lens designs, which are based on manipulating peripheral vision and have an asymmetrical design. The refractive power of the peripheral gradually increases in relative positive power to achieve peripheral myopic defocus on the peripheral. This defocus signal sent to the eye is believed to affect eye growth, effectively slowing the progression of myopia. Stellest lenses (spectacle lens with highly aspherical lenslets [HAL]) are a recent myopia control lens design, which are multifocal spectacle lenses with highly aspherical lenslets. The front surface is spherical and consists of 11 concentric rings formed by adjacent highly aspherical lenslets (1.1 mm in diameter). These aspherical lenslets deviate rays of light continuously in a nonlinear manner that creates a three-dimensional quantity of light in front of the retina, which we call volume of myopic defocus. Several studies have shown greater asphericity; a larger volume of myopic defocus could more effectively slow the progression of myopia.^8^^,^^18^^,^^19^

The effectiveness of PDSL in controlling myopia has been confirmed, but most previous studies have focused primarily on changes in axial length and spherical equivalent refraction (SER) without further analyzing their potential impact on astigmatism progression. Astigmatism consists of corneal astigmatism and intraocular astigmatism.^20^ The cornea's shape and curvature are direct determinants of astigmatism. Intraocular astigmatism mainly comes from the lens, which exerts a comparatively minor and consistent influence on astigmatism.^21^ As myopia progresses, the elongation of the eyeball's posterior pole could, in turn, impact the anterior segment of the eye. This may alter the position and curvature of both the cornea and the lens.^22^ Clinical cases from the Eye Hospital of Wenzhou Medical University have observed notable astigmatism progression in some myopic children after PDSL intervention. The increase in astigmatism not only simultaneously leads to a visual acuity deficit^23^ but also decreases the effectiveness of myopia control, because the rise in astigmatism adds to the amount of SER. Furthermore, a study has shown that astigmatism negatively impacts the myopia treatment outcomes of PDSL.^24^

It is currently unknown whether the increase in astigmatism is occasional and reversible or a common phenomenon that could become permanent. This study aims to uncover the impact of wearing PDSL on cylindrical refractive error (CYL) of myopic children and the mechanisms behind these effects.

## Methods

### Study Design

This study was a retrospective design study. Medical records were acquired through the outpatient medical record system of the Eye Hospital of Wenzhou Medical University (WMU). This study enrolled myopic children and adolescents aged six to 17 years at the Eye Hospital of WMU from February 2003 to December 2022. Participants who were fitted PDSL were included in the study group, while the control group consisted of participants who either did not wear glasses or only wore single-vision frame glasses without any myopia control measures. Inclusion criteria include (1) SER between −0.50 D and −10.50 D; (2) at least two optometry records with a time interval of at least one year (the first optometry record for the study group was taken at the initial fitting of PDSL); and (3) best-corrected visual acuity ≥0.1 logarithm of the minimum angle of resolution. Exclusion criteria include (1) any applications of myopia prevention and control measures other than PDSL, such as low-concentration atropine eye drops, orthokeratology, multifocal soft lenses, and more and (2) presence of any other eye diseases, such as congenital cataracts, glaucoma, retinopathy, and more.

All procedures in this study adhered to the principles of the Helsinki Declaration. The research protocol was reviewed and approved by the Ethics Review Committee of the Eye Hospital of Wenzhou Medical University (Approval No: 2022-108-K-82). Because of the retrospective design, the study was granted a waiver of informed consent by the Ethics Review Committee.

### Samples and Groups

**Figure 1.**
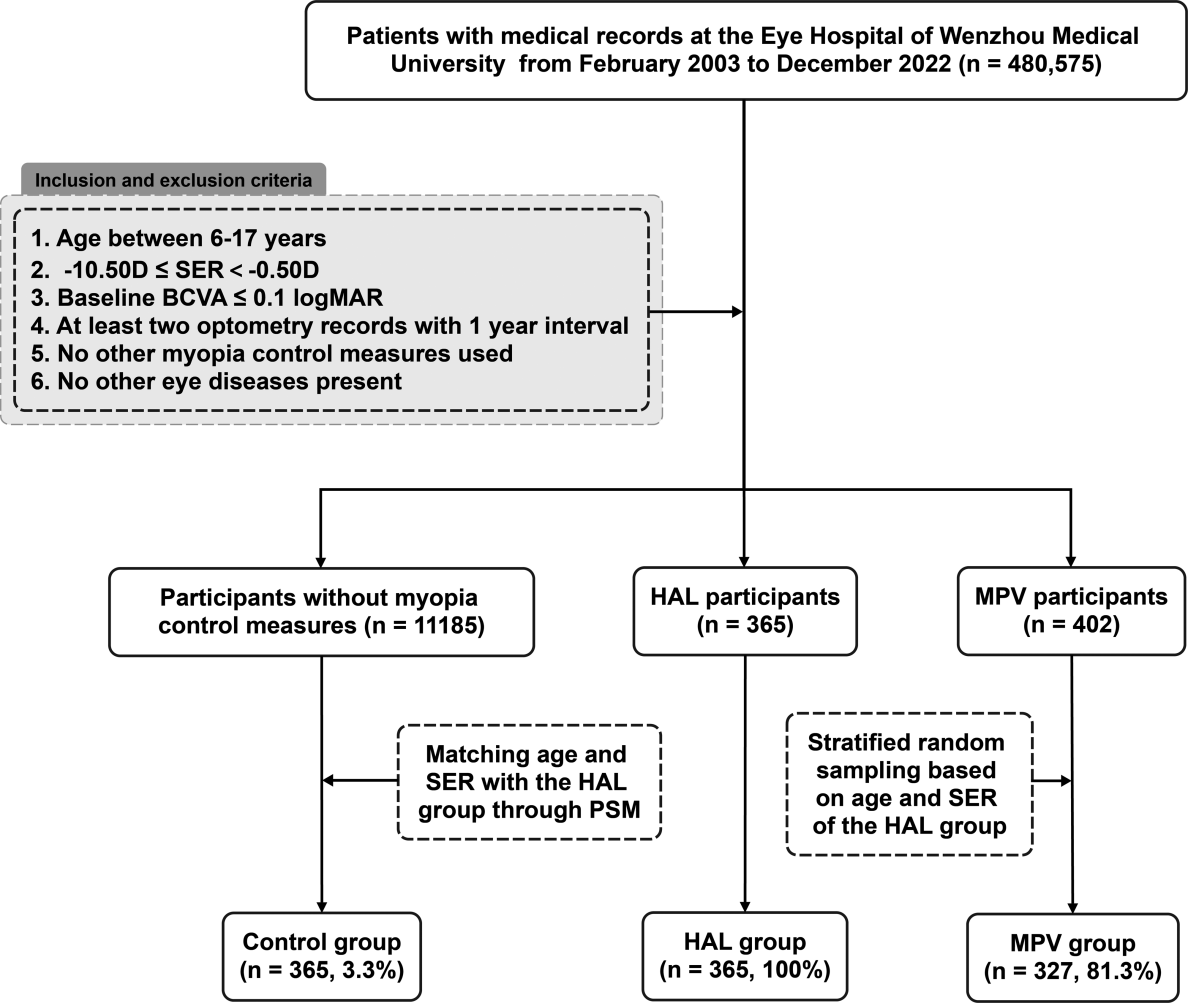
Flow chart of samples. BCVA, best corrected visual acuity; logMAR, logarithm of the minimum angle of resolution; PSM, propensity score matching.

The Eye Hospital of WMU primarily uses two types of PDSL fittings—Stellest lens (HAL) and Myovision lens (MPV)—which are also widely used across China. The participants in the control group were matched with HAL in terms of age and SER through propensity score matching analysis, with a matching ratio of 1:1. A matching tolerance of 0.04 was set by trial-and-error approach to achieve the maximum sample size. Because good baseline characteristic matching between the HAL group and the MPV group could not be achieved through propensity score matching, the MPV group was hierarchically matched and stratified with the HAL group according to the baseline age and baseline SER. Based on the number of individuals in each stratum of the HAL group, cases in the MPV group were randomly selected in the same proportion to match the baseline characteristics of the HAL group (Fig. 1).

### Statistical Analysis

The present study is interested in participants’ annual change in SPH, CYL, and SER. SER was calculated as the sum of SPH and half of the CYL based on subjective refraction. CYL represents total astigmatism (TA). In addition, this study also examines the consistency between CYL change and corneal astigmatism (CA) change, with CA being the difference in refractive power between the two principal meridians of the cornea (i.e., the flattest keratometry value and the steepest keratometry value) as measured by the IOL MASTER. Baseline characteristics include baseline age, baseline SER, gender, and baseline CYL. Continuous variables were described using “mean ± standard deviation” or “median (interquartile range).” Categorical data were expressed with frequency and percentage. SPSS (Chicago, IL, USA, version 27) was used for statistical analysis.

This study only analyzed data from the right eye, with a significance level set at 0.05. The Kruskal-Wallis test and Mann-Whitney U test were used to analyze the differences of baseline characteristics, as well as changes in SER, SPH, and CYL among groups. Baseline characteristics with intergroup differences were further analyzed for their correlations with changes in SER, SPH, and CYL using Spearman correlation analysis. Wilcoxon signed-rank test was used to analyze differences between CYL change and CA change. Additionally, the Bland-Altman plot was used to assess the consistency between CYL change and CA change. The χ^2^ test was used to assess proportions. Linear regressions were used to analyze relations. Mediation analysis, which is commonly used to evaluate whether an intervention affects outcomes through causal mechanisms, was conducted in this study to assess the potential effect of SPH on CYL change. The mediation effect and confidence intervals were tested using the bootstrap method, and results were considered statistically significant if the confidence interval (CI) did not include 0.

## Results

**Figure 2.**
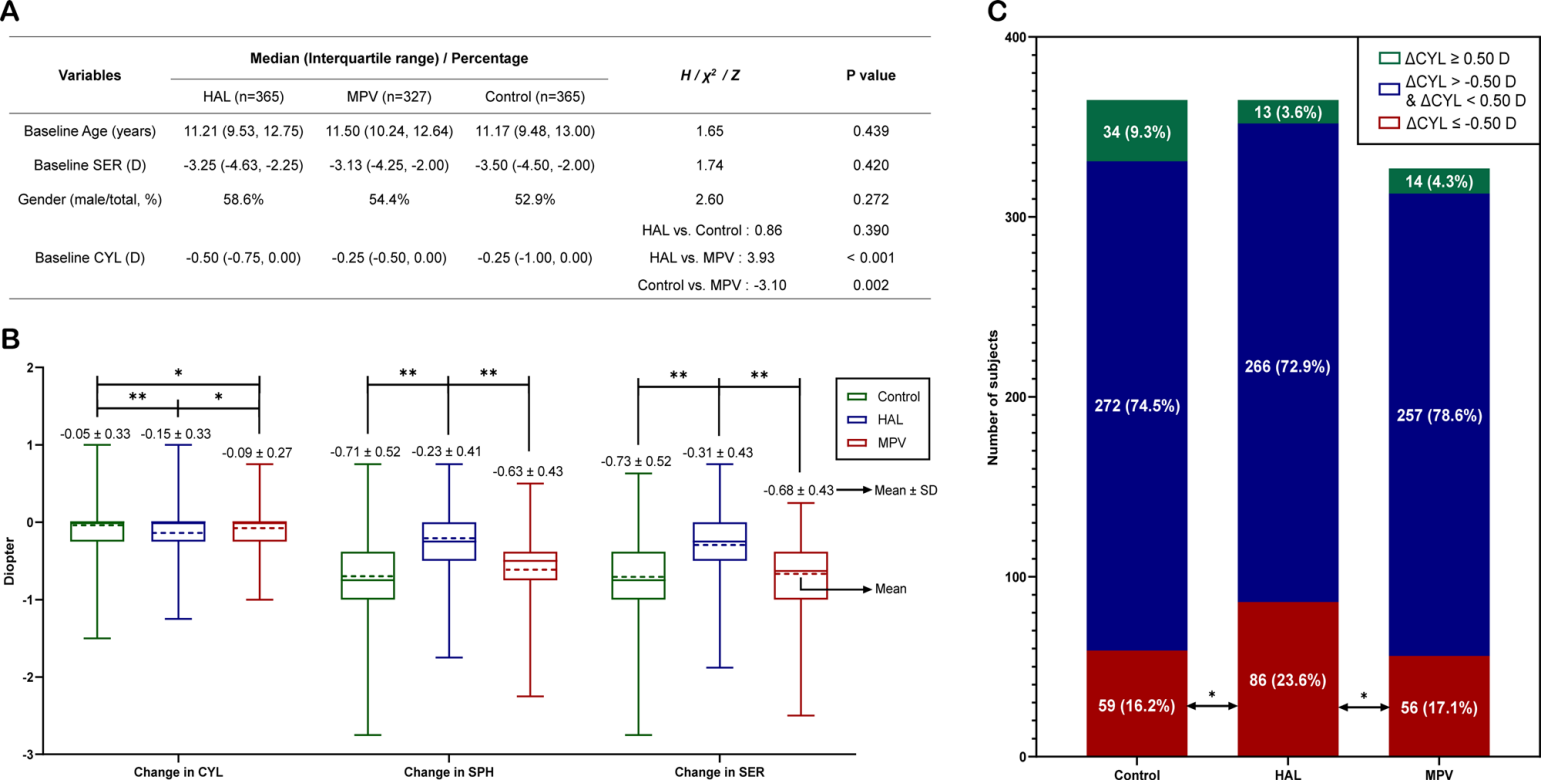
Baseline characteristics and refractive error changes in each group. (**A**) Baseline characteristics of each group. (**B**) Change of refractive errors within one year for each group. In the box plots, the *solid line* represents median, the *dashed line* represents mean, and the *error bars* represent interquartile range. Mean ± SD is indicated at the top of each box plot. (**C**) Proportions of one-year change of cylindrical refractive error in each group. ** *P* < 0.001; * *P* ≥ 0.001. ΔCYL ≤ −0.50 D, subjects with CYL increase ≥0.50 D within one year; ΔCYL ≥ 0.50 D, subjects with CYL decrease ≥0.50 D within one year; SD, standard deviation.

This study involved 1057 myopic participants from the Eye Hospital of WMU, including 365 in the HAL group, 327 in the MPV group and 365 in the control group. Except for the MPV group having a smaller baseline CYL compared to the other two groups, no significant differences in baseline characteristics were found among the three groups (Fig. 2A). However, there was no significant correlation between baseline CYL and changes in CYL, SPH, or SER over one year in each group (all *P* > 0.05, Supplementary Table S1).

### Change in CYL

**Figure 3.**
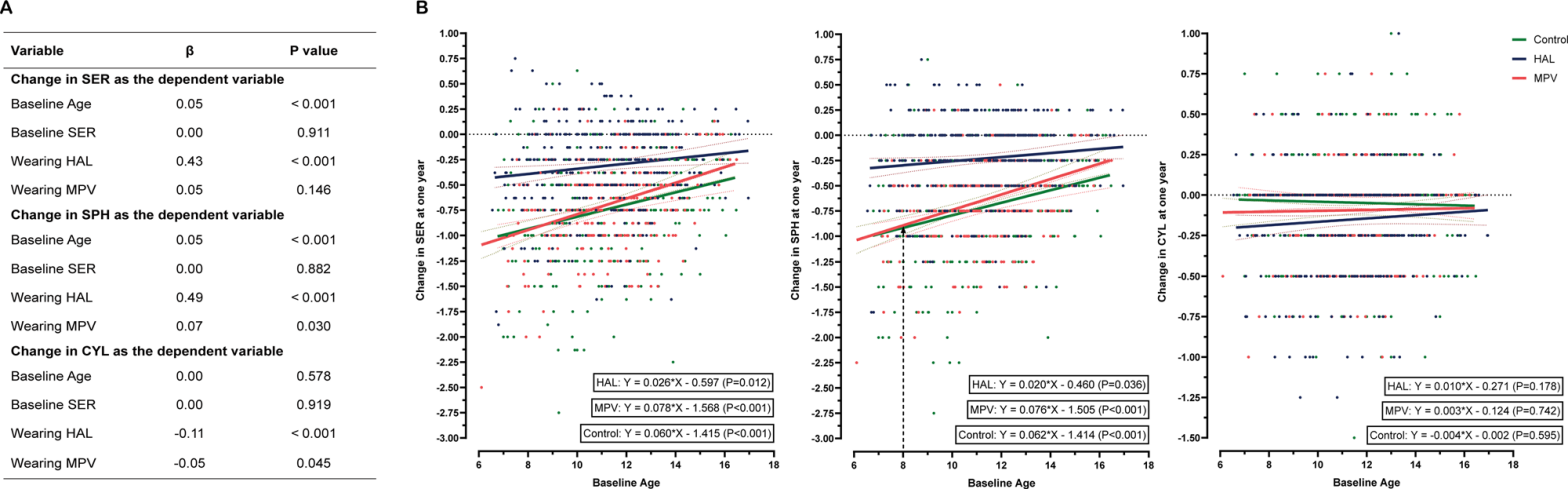
Linear regression analysis of factors influencing the change of refractive error over one year. (**A**) The effect of wearing peripheral defocus–designed spectacle lenses on refractive error. Three linear regression models analyzed the impact of wearing HAL or MPV on the annual change in refractive error with age, baseline SER corrected. (**B**) The age effect on refractive error. Scatter plots and regression lines show the relationship between age and one-year change of refractive error in each group.

The average one-year change of CYL was −0.15 ± 0.33 D in the HAL group, −0.09 ± 0.27 D in the MVP group, and −0.05 ± 0.33 D in the control group, with significant CYL progression observed in all groups over one year (HAL: *P* < 0.001; MPV: *P* < 0.001; Control: *P* = 0.008). The CYL in the HAL group showed the largest progression, followed by the MPV group, and the control group (HAL vs. MPV: *P* = 0.023; HAL vs. Control: *P* < 0.001; MPV vs. Control: *P* = 0.019, Fig. 2B). The proportion of participants with annual CYL change greater than or equal to 0.50 D was 23.6% (86/365) in the HAL group, 17.1% (56/327) in the MPV group and 16.2% (59/365) in the control group. The proportion in the HAL group was 38.0% higher than that in the MPV group (*P* = 0.036) and 45.7% higher than that in the control group (*P* = 0.012). The proportion in the MPV group was 5.6% higher than that in the control group (*P* = 0.735, Fig. 2C). Multiple linear regression analysis also revealed that, compared to the control group, wearing HAL (β = −0.11, *P* < 0.001) significantly accelerated CYL progression, whereas MPV (β = −0.05, *P* = 0.045) had a milder effect on accelerating CYL progression (Fig. 3A), with age and baseline SER corrected. Age and baseline SER did not affect CYL change (Fig. 3A). Among the 209 participants in the HAL group and 140 participants in the MPV group with baseline astigmatism, no significant changes in the cylinder axis were observed after wearing PDSL for one year (HAL: *P* = 0.422, MPV: *P* = 0.421).

### Change in SPH

The average one-year change of SPH in HAL, MPV, and control groups was −0.23 ± 0.41 D, −0.63 ± 0.43 D, and −0.71 ± 0.52 D, respectively. The change of SPH in the HAL group was significantly smaller than that in the other two groups (HAL vs. MPV: *P* < 0.001; HAL vs. Control: *P* < 0.001, Fig. 2B). Multiple linear regression analysis also revealed that, compared to the control group, wearing HAL (β = 0.49, *P* < 0.001) significantly slowed SPH progression, whereas wearing MPV (β = 0.07, *P* = 0.030) also mildly slowed SPH progression, with age and baseline SER corrected (Fig. 3A). Age-based linear regressions within each group showed that as age increased, the SPH change in the HAL group remained stable and consistently smaller than in the other two groups, while the SPH change in the MPV and control groups decreased over time, with the MPV group experiencing a greater rate of decrease compared to the control group (β = 0.08 for the MPV group, β = 0.06 for the control group, Fig. 3B). The SPH change difference between the MPV and the control group became significant after age 8 (MPV vs. control: −0.61 ± 0.41 D vs. −0.70 ± 0.51 D, *P* = 0.018).

### Change in SER

The average one-year change of SER was −0.31 ± 0.43 D, −0.68 ± 0.43 D, and −0.73 ± 0.52 D in the HAL, MPV and control groups, respectively. The SER change in the HAL group was significantly smaller than that of the other two groups (HAL vs. MPV: *P* < 0.001; HAL vs. Control: *P* < 0.001). There was no significant difference in SER progression between the MPV group and the control group (*P* = 0.431, Fig. 2B). Multifactorial linear regression analysis revealed that wearing HAL (β = 0.43, *P* < 0.001) significantly decreased SER change compared to the control group whereas wearing MPV (β = 0.05, *P* = 0.146, Fig. 3A) had no significant impact on the SER change compared to the control group. The SER change in older participants was smaller than in younger participants (β = 0.05, *P* < 0.001). Age-based linear regression analysis in each group showed that as age increases, SER change in the HAL group was stable and consistently smaller compared to the other two groups. In contrast, the SER change in the MPV group and the control group decreased as age increased (Fig. 3B).

### The Potential Origins of Cylindrical Refractive Error Change

The causal relationship between CYL change and SPH change in the HAL group and MPV group can be presented through mediation analysis (Table). The total impact of wearing HAL and MPV on CYL change revealed by mediation analysis was −0.11 (95% confidence interval [CI], −0.15 to −0.06) and −0.05 (95% CI, −0.09 to 0.00), respectively (Table), which was consistent with the coefficients from linear regressions (Fig. 3A). Compared to the control group, the CYL change in the HAL group can be explained by two pathways: direct induction through wearing HAL (β = −0.07, 95% CI, −0.11 to −0.02) as a primary pathway, and indirect induction through slowing the progression of SPH (β = −0.04, 95% CI, −0.06 to −0.02) as a complementary pathway. Similarly, in the MPV group, the acceleration of CYL progression could also be explained by direct and indirect induction, but these results were not statistically significant.

**Table.** The Effect of Wearing Peripheral Defocus Designed Spectacle Lenses on Cylindrical Refractive Error Changes

		95% CI	
Pathway (Compared With Control Group)	Coefficients	Lower CI	Upper CI
HAL group			
Total effect: HAL → Change in CYL	−0.11^*^	−0.15	−0.06
Direct effect: HAL → Change in CYL	−0.07^*^	−0.11	−0.02
Indirect effect: HAL → Change in SPH → Change in CYL	−0.04^*^	−0.06	−0.02
MPV group			
Total effect: MPV → Change in CYL	−0.05	−0.09	0.00
Direct effect: MPV → Change in CYL	−0.04	−0.09	0.01
Indirect effect: MPV → Change in SPH → Change in CYL	−0.01	−0.02	0.00

CI, confidence interval; Change in SPH, one-year change in spherical refractive errors; Change in CYL, one-year change in cylindrical refractive errors.

* Coefficients were statistically significant.

**Figure 4.**
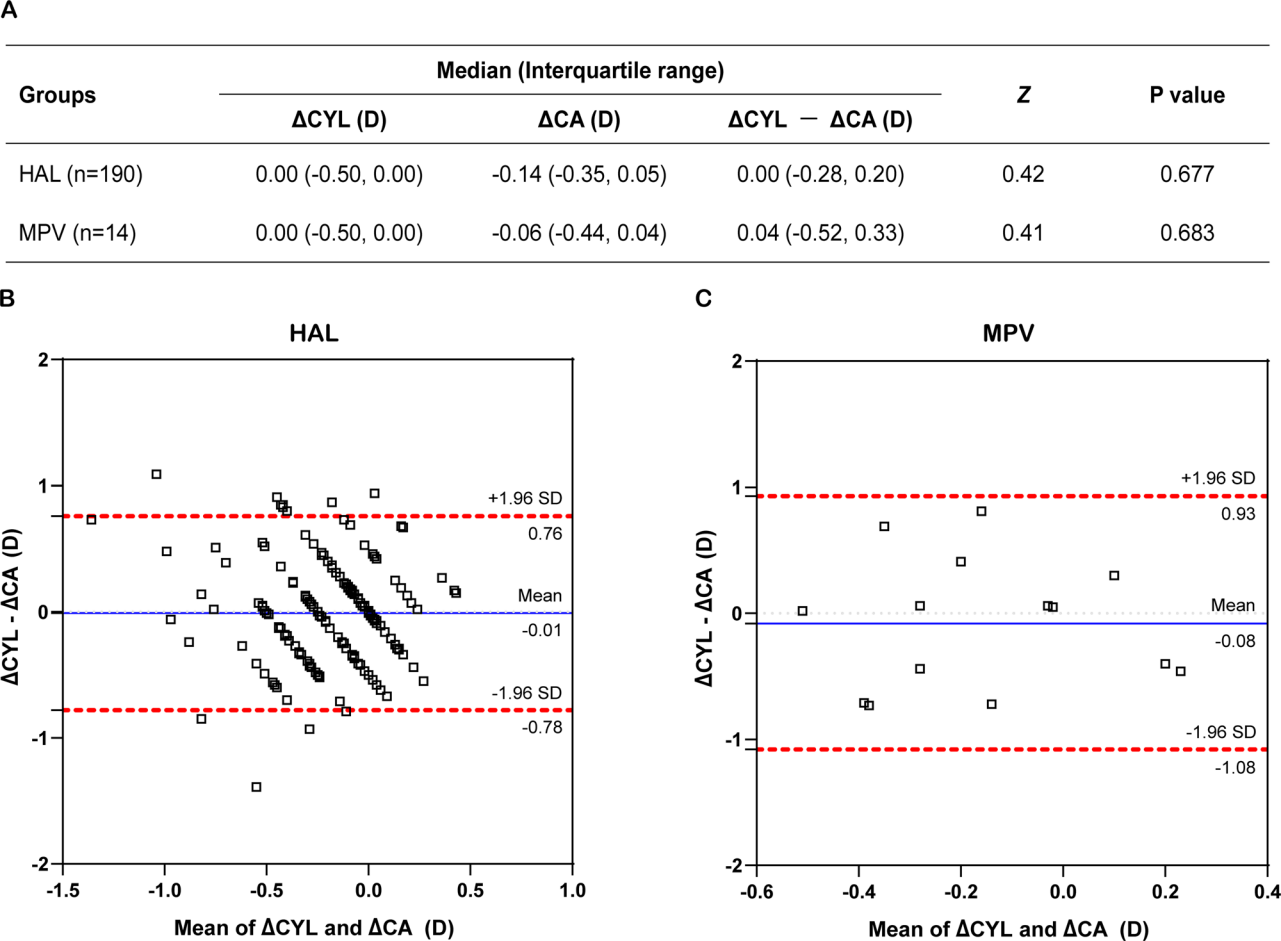
Differences and consistency between ΔCYL and ΔCA in the HAL group and MPV group. (**A**) Differences between ΔCYL and ΔCA within each group. (**B**) Bland-Altman plot showed the consistency between ΔCYL and ΔCA in HAL group. (**C**) Bland-Altman plot showed the consistency between ΔCYL and ΔCA in MPV group. The *blue solid line* in the Bland-Altman plots represent the mean difference between ΔCYL and ΔCA, whereas the *red dashed lines* indicate the 95% limits of agreement. Most of the dots fall within the dashed boundary, which means good consistency of two variables. ΔCYL, one-year change in cylindrical refractive error; ΔCA, one-year change in corneal astigmatism; ΔCYL − ΔCA, the difference between the one-year change in cylindrical refractive error and the one-year change in corneal astigmatism.

Among the myopic children included in this study, 190 out of those who were fitted HAL underwent IOL master measurements, and 14 out of those who were fitted MPV did so. No significant differences were observed in the comparisons of CA changes and CYL changes in the HAL (*P* = 0.677) group and the MPV (*P* = 0.683) group (Fig. 4A). Further analysis using Bland-Altman plots showed good consistency between the CYL change and the CA change in both the HAL group and the MPV group. In the HAL group, the limit of agreement (95% CI) for the difference between the CYL change and the CA change was −0.78 D to 0.76 D, with 93.7% (178/190) of the participants falling within this range (Fig. 4B). In the MPV group, the limit of agreement for the difference was −1.08 D to 0.93 D, with 100% (14/14) of participants falling within this range (Fig. 4C). These indicated that CA and CYL are relatively changed synchronously within both groups.

## Discussion

### Changes in Astigmatism

Significant astigmatism progression was observed in participants wearing PDSL over one year. The percentage of participants who experienced an astigmatism increase ≥0.50 D was higher in the HAL group compared to the controls.

Mediation analysis indicated that wearing PDSL could directly and indirectly accelerate the progression of astigmatism. The direct impact was wearing HAL or MPV themselves, and the indirect impact was induced by the myopia control effect. Both wearing HAL or MPV could indirectly accelerate the progression of astigmatism by delaying the progression of SPH. The increase in astigmatism caused by wearing HAL was greater than that caused by wearing MPV. It was speculated that HAL had a better control effect on SPH progression than MPV and thus had a greater indirect impact. The increase of astigmatism in the MPV group was not significant in the meditation analysis. This might be because the CYL change was very small, and mediation analysis cannot show its efficiency because of high variability.

Although the present study is the first one to report astigmatism changes after wearing PDSL, previous animal experiments had demonstrated parallel results to the present study. Interventions in the natural visual development of chicks or monkeys^25^^–^^27^ and convex lens–induced myopia models could also induce astigmatism progression.^25^ Together, these results implicate that myopia and astigmatism might interact when visual or optical inputs intervened.

This study also revealed that the progression of astigmatism after wearing PDSL primarily originated from CA progression, which can be partially explained by PDSL's effect on controlling SPH progression. Coupled with previous research indicating that wearing PDSL significantly delays SPH progression and axial length elongation^8^^,^^9^^,^^19^ and even increases the choroid thickness,^28^^,^^29^ it is speculated that changes in ocular fundus structure induced by visual intervention may influence the biomechanics of the anterior segment of the eye.^30^ The possible mechanisms are as follows: First, previous studies have found that altering the visual conditions could result in changes in corneal shape. This is akin to passive outcomes during “emmetropization,” where the eye adjusts the scleral extracellular matrix based on retinal image focus, influencing anterior ocular biomechanics while controlling axial length elongation^26^^,^^27^^,^^31^^,^^32^; second, PDSL primarily controls posterior axial length elongation by forming myopic defocus signal zones in the peripheral retina, thereby regulating SPH progression. This means that PDSL exerts a forward traction on the eye, which may lead to increased pressure between the anterior surface of the eyeball and the eyelid, resulting in the progression of CA.^33^^–^^35^ Additionally, the optical aberrations produced by wearing PDSL may also induce changes in the curvature of the cornea or lens, leading to an increase in astigmatism.^36^^–^^38^ This phenomenon of astigmatism progression was also noted in a study on 0.01% atropine eye drops, where atropine effectively controlled myopia progression while simultaneously causing astigmatism progression, primarily related to CA.^39^

In summary, the origin of astigmatism progression after wearing PDSL may be mainly attributed to changes in corneal morphology, whereas the mechanism of CA reshaping cannot be directly determined from the results and requires further investigation. It can be confirmed that no axis change was found after wearing HAL or MPV, indicating that the passive changes in CA primarily reflect changes in corneal curvature, without leading to irregular development.

### Myopia Control Effect

In this study, myopic participants wearing HAL showed an average SER progression of −0.31 D over one year, which was similar with results reported by Tang et al.^40^ and Guo et al.^41^ The study also found that HAL could slow the progression of SER by an average of 0.42 D per year compared to the control group, which is consistent with studies conducted by Bao et al.^19^ and Tang et al.^40^ The average annual SPH change in the three groups of this study was smaller than the average annual SER change, which may have been influenced by the increase in negative astigmatism.

Previous research indicated that the short-term myopia control effect of MPV was not ideal,^14^^,^^15^ but HAL effectively controls myopia.^5^^,^^8^^,^^15^^,^^18^^,^^19^^,^^23^^,^^30^^,^^42^ This study implies that astigmatism progression, lens type, and age would influence the effectiveness of PDSL in myopia control. Astigmatism progression reduced the effectiveness of PDSL in myopia control. Although MPV could slow the progression of SPH, it simultaneously promoted an increase in astigmatism progression. This might result in an insignificant difference in the change of SER over one year. This study also found that in both the HAL and MPV groups, older myopic children experienced better myopia control effectiveness. Similar findings were observed in the use of DIMS.^43^

However, limitations exist. First, the results of this study were obtained based on a one-year observation period. Future studies should focus on the long-term impact of wearing PDSL on astigmatic progression. Second, the current study includes participants observed over a 20-year time span, during which societal, economic, cultural, and lifestyle factors may have changed. Although this study addressed this issue by controlling and matching baseline characteristics, the potential bias should not be ignored. Third, the MPV group has a relatively small number of participants with IOL Master records, which may introduce potential inaccuracy in the consistency analysis of the relationship between corneal astigmatism and total astigmatism.

## Conclusions

Wearing periphery defocus lenses could induce an increase in astigmatism, both directly and indirectly, with changes primarily originating from the cornea.

## Supplementary Material

Supplement 1
