# Supplementary material for: The Peripheral Defocus Designed Spectacle Lenses Might Increase Astigmatism in Myopic Children
Source: Transl Vis Sci Technol. 2025 Mar 11;14(3):8. doi: 10.1167/tvst.14.3.8 (PMC11918031; doi:10.1167/tvst.14.3.8)
Supplement: Supplement 1 [file tvst-14-3-8_s001.docx]

| **Supplementary Table S1. The correlation between baseline CYL and changes of refractive errors within one year in each group** | | | | |
| --- | --- | --- | --- | --- |
| **Groups** | | **Change in CYL** | **Change in SPH** | **Change in SER** |
| Control | r | -0.063 | -0.073 | -0.102 |
|  | P value | 0.230 | 0.166 | 0.053 |
| HAL | r | -0.042 | 0.072 | 0.040 |
|  | P value | 0.441 | 0.180 | 0.453 |
| MPV | r | -0.123 | -0.180 | -0.225 |
|  | P value | 0.347 | 0.168 | 0.083 |
| Analyzed using Spearman correlation analysis.  HAL, spectacle lens with highly aspherical lenslets; MPV, spectacle lens based on manipulating peripheral vision; CYL, cylindrical refractive errors, all data are recorded in negative cylinder format; SPH, spherical refractive error; SER, spherical equivalent refraction. | | | | |
